# Role of IgA1 protease-producing bacteria in SARS-CoV-2 infection and transmission: a hypothesis

**DOI:** 10.1128/mbio.00833-24

**Published:** 2024-08-29

**Authors:** Michael W. Russell, Mogens Kilian, Jiri Mestecky

**Affiliations:** 1 Department of Microbiology and Immunology, Jacobs School of Medicine and Biomedical Sciences, University at Buffalo, Buffalo, New York, USA; 2 Department of Biomedicine, Aarhus University, Aarhus, Denmark; 3 Department of Microbiology, Heersink School of Medicine, University of Alabama at Birmingham, Birmingham, Alabama, USA; ^4^Institute of Microbiology, laboratory of Cellular and Molecular Immunology, Czech Academy of Sciences, Prague, Czechia; University of Illinois Chicago, Chicago, Illinois, USA

**Keywords:** COVID-19, SARS-CoV-2, mucosal immunity, immunoglobulin A, bacterial IgA1 protease

## Abstract

Secretory (S) IgA antibodies against severe acute respiratory syndrome (SARS)-CoV-2 are induced in saliva and upper respiratory tract (URT) secretions by natural infection and may be critical in determining the outcome of initial infection. Secretory IgA1 (SIgA1) is the predominant isotype of antibodies in these secretions. Neutralization of SARS-CoV-2 is most effectively accomplished by polymeric antibodies such as SIgA. We hypothesize that cleavage of SIgA1 antibodies against SARS-CoV-2 by unique bacterial IgA1 proteases to univalent Fabα antibody fragments with diminished virus neutralizing activity would facilitate the descent of the virus into the lungs to cause serious disease and also enhance its airborne transmission to others. Recent studies of the nasopharyngeal microbiota of patients with SARS-CoV-2 infection have revealed significant increases in the proportions of IgA1 protease-producing bacteria in comparison with healthy subjects. Similar considerations might apply also to other respiratory viral infections including influenza, possibly explaining the original attribution of influenza to *Haemophilus influenzae*, which produces IgA1 protease.

## OPINION/HYPOTHESIS

Severe acute respiratory syndrome (SARS)-CoV-2 is an airborne virus that first infects the human upper respiratory tract (URT; nasal passages and pharynx) by the inhalation of aerosols and droplets, or possibly also by contact with the conjunctiva of the eye followed by drainage into the nasal passages through the lacrimal duct (1). Although a significant proportion of individuals, possibly up to 50%, experience only mild disease or even remain asymptomatic, the descent of the virus into the lower respiratory tract (LRT; bronchial tree and terminal airways) can result in the SARS known as COVID-19, characterized by dysregulated inflammatory responses with life-threatening consequences. As would be expected of infections that arise at mucosal surfaces, many studies have now demonstrated that SARS-CoV-2 induces mucosal immune responses revealed by the generation of secretory IgA (SIgA) antibodies in nasal, oral, and other secretions against viral antigens (reviewed in 1, 2–6). Furthermore, studies of nasal or salivary IgA antibodies to SARS-CoV-2 antigens in relation to the course of infection have shown that promptly and strongly developed mucosal IgA responses were associated with more rapid clearance of the virus and resolution of symptoms (7, 8). In adults challenged intranasally with live SARS-CoV-2, prompt mucosal IgA (as well as CD8+ T cell) responses were strongly associated with viral control (9). Thus, it is now increasingly recognized that mucosal IgA antibody responses are likely critical in controlling initial infection with SARS-CoV-2 and hence determining the subsequent clinical outcome (10). The injectable vaccines that have been successfully developed to control the pandemic induce systemic immune responses represented largely by circulating IgG antibodies and T cells, which have been credited with clearing the virus from the LRT, alleviating the disease and its lethal consequences (1). However, these vaccines do not effectively induce SIgA antibody responses in naïve individuals, although there is evidence that they may help to recall SIgA antibodies in subjects who were previously infected with SARS-CoV-2 (11).

SARS-CoV-2 is spread from infected subjects by means of droplets and aerosols emitted during normal breathing and speech, and exacerbated by sneezing, coughing, vigorous breathing associated with physical exercise, or shouting and singing. The source of these droplets and aerosols lies in the mucosal secretions of the URT and the mouth (saliva) (12), but these are not merely passive vehicles of transmission. Infectivity of these emissions will be determined by the load of infectious viral particles generated by the replication of the virus within the mucosal epithelia and the infectious characteristics of the particular viral strain, but counteracted by the virus-neutralizing activity of the secretions. The latter consists largely of neutralizing anti-viral antibodies, predominantly SIgA, the major immunoglobulin isotype present in saliva and URT secretions (Table 1).

**TABLE 1:** Comparative properties and differences between mucosal (nasal and oral) and circulating immunoglobulins^*f*^

	Nasal	Oral	Blood (plasma)
Concentrations (µg/mL):			
IgG	8–304^*a*^	1–42^*b*^	7,000–13,000
IgM	?^*c*^	64	500–2,500
IgA	70–846	194–206	500–3,500
IgA subclasses (%):			
IgA1	93^*d*^	63	85
IgA2	7^*d*^	37	15
IgA molecular forms (%):			
pIgA	?^*e*^	96	1–5
mIgA	?^*e*^	4	95–99
Sites of IgA production	Nasal mucosa	Salivary glands	Bone marrow (spleen, lymph nodes)

^*a*^ Dependent on method of collection.

^*b*^ Dependent on gingival crevicular fluid.

^*c*^ Not reported.

^*d*^ Based on % of IgA1- or IgA2-secreting cells.

^*e*^ Not established.

^*f*^ Modified from ref. (1) (© Russell and Mestecky).

Susceptibility to COVID-19 disease varies between individuals due to a variety of factors that are incompletely understood. However, as noted above, several studies have revealed that the early and robust development of mucosal IgA antibody responses in children and adults is associated with rapid elimination of the virus and control of symptomatic disease (7–9). Some individuals infected with SARS-CoV-2 appear to be more effective than others in transmitting the virus; such individuals have been designated “superspreaders” (13, 14). Superspreading capability is undoubtedly due to multiple factors, including behavioral characteristics and environmental conditions, but that will also depend on the infectious load of virus and on virus-neutralizing antibody activity in the secretions.

SARS-CoV-2 has been shown to be neutralized most effectively by antibodies against the spike protein, which is responsible for initial binding and uptake of the virus by the ACE2 receptor on epithelial cells (15). While any antibody isotype of the right specificity can neutralize viruses, polymeric (p) IgA antibodies have long been known to have superior neutralizing capability against viruses such as influenza, compared to monomeric (m) IgA or IgG antibodies (16, 17). This has recently been confirmed for SARS-CoV-2 with engineered monoclonal antibodies having the same antigen-binding variable domains but different constant domains (IgG vs IgA) and with IgA in dimeric (J chain-containing) or monomeric configuration; dimeric IgA antibodies showed 15- to 240-fold greater neutralizing activity relative to mIgA or IgG (18, 19). An IgG monoclonal antibody engineered to be hexameric also demonstrated greatly increased viral neutralizing activity compared to its monomeric form (20). Thus, the neutralizing activity increases markedly with the polymeric molecular form of antibodies, likely due to their enhanced functional avidity arising from multivalent antigen binding, coupled with greater steric hindrance of the bound antigen due to the greater molecular bulk of polymeric antibodies. It therefore logically follows that the much smaller, univalent Fab fragments of antibodies should have substantially reduced virus-neutralizing capacity compared to intact antibodies especially those of polymeric form, even though Fab fragments retain their antigen-binding activity and intrinsic affinity. However, to our knowledge, the virus-neutralizing capacity of antibody Fab fragments has not been examined. Fab fragments also cannot mediate other defense mechanisms such as agglutination, complement activation, phagocytosis, and the TRIM-21 pathway of intracellular inhibition of viral replication by non-neutralizing antibodies (or in the case of SIgA, trapping in the mucus layer), which require sequence motifs present in the Fc regions (and secretory component in SIgA) (21, 22). In contrast to the dominant mIgA form present in plasma, SIgA1 in secretions is found mainly in dimeric and tetrameric configurations with four or eight antigen-binding sites. The presence of multiple binding sites in pIgA greatly enhances (by one to two orders of magnitude) its biological functions, such as virus neutralization, relative to mIgA. The “bonus effect of multivalency” in various biological functions of antibodies has been amply demonstrated in numerous studies of mIgA or IgG in comparison with pIgA or IgM.

In humans, chimpanzees, gorillas, and gibbons, IgA occurs in two subclasses, IgA1 and IgA2, which display a characteristic distribution in various body fluids (23). In contrast, other mammals have IgA that more closely resembles human IgA2 (24). IgA1 is dominant in human plasma, saliva, and URT secretions while IgA2 prevails in the secretions of the large intestine and female reproductive tract. The most important structural difference between IgA1 and IgA2 lies in the hinge region, which in IgA1 is 19 residues in length and includes a repeat sequence of 8 amino acids containing several proline, serine, and threonine residues and ~6–8 O-linked glycans (24). In contrast, the IgA2 hinge region is six residues in length, of which five are prolines. Because of its unusual amino acid sequence and abundant glycosylation, the hinge region of IgA1 is highly resistant to proteolytic attack but is the target of a group of unique bacterial IgA1-cleaving proteases resulting in the generation of Fab and Fc fragments; these enzymes are discussed below. Although IgA subclass responses in COVID have not been well investigated, at least one study has shown these antibodies to be mainly IgA1, as expected in saliva and URT secretions, and for anti-viral IgA antibody responses (25).

The humoral components of the mucosal immune system operate in an enzymatically hostile environment. Bacteria in the microbiota of the mucosal tracts release an array of proteases and glycosidases that compromise the functional integrity of immunoglobulins and complement factors (26). Only SIgA is known to remain largely intact during the entire gastrointestinal passage; the secretory component of SIgA has been shown to afford substantial protection against common proteases (27, 28). However, a group of functionally similar bacterial IgA1 proteases generated by diverse bacterial species specifically cleave a single post-proline peptide bond (Pro-Thr or Pro-Ser) in the hinge region of human IgA1 (including SIgA1), but not IgA2 (reviewed in 26, 29). Notably, these IgA1 proteases are produced by all three principal causes of bacterial meningitis, *Streptococcus pneumoniae, Haemophilus influenzae* serotype b, and *Neisseria meningitidis*; in *S. pneumoniae* a metalloprotease (ZmpA) and in the two Gram-negatives a serine-type endopeptidase (IgA). In addition to causing invasive infections in humans, all three pathogens are commonly carried in the nasopharynx of healthy individuals. Likewise, the commensals, non-type b *H. influenzae, Haemophilus parahaemolyticus*, and several commensal species of *Streptococcus* (*Streptococcus mitis*, *Streptococcus oralis*, and *Streptococcus sanguinis*) and *Gemella*, which are dominant members of the microbiota of the URT and mouth, produce an IgA1 protease. In addition, species of *Capnocytophaga* and *Prevotella* produce IgA1 proteases (30). The monomeric Fabα fragments released by IgA1 proteases retain their antigen-binding activity but lose the biological functions associated with Fcα (31). Furthermore, the Fabα fragments may competitively interfere with the binding of Igs of other isotypes and thereby block their protective activities such as complement-activation and opsonization (26). There is evidence that monomeric Fabα fragments of specific IgA antibodies generated *in vivo* facilitate mucosal adherence and cellular invasion of IgA1 protease-producing pathogens (32, 33). Fabα fragments of specific IgA antibodies have been identified in the secretions of individuals harboring IgA1 protease-producing bacteria (30, 34, 35). The extent of SIgA1 cleavage may be assumed to depend on the relative proportions of IgA1-protease producing bacteria in the local microbiota. In contrast, while Fab fragments of antibodies, especially IgG, can be readily generated *in vitro*, there is no normal source of such fragments *in vivo*.

## THE HYPOTHESIS

It is therefore reasonable to propose that, in SARS-CoV-2-infected subjects who carry IgA1 protease-producing bacteria in their mouths or URT, SIgA1 antibodies against SARS-CoV-2 in these secretions would be susceptible to cleavage to much less effective Fabα (and Fcα) fragments. This would diminish their ability to neutralize the virus, which in turn might not only increase the possibility of the virus descending into the LRT and causing serious COVID disease, but also enhance their ability to infect others, that is, render them “superspreaders” especially when other factors combine to enhance this effect. This should be a testable hypothesis, although doing so would require considerable effort and resources, as for all practical purposes, it could only be accomplished in human studies. Note that, apart from great apes and gibbons, no other mammalian species possess human-like IgA1, effectively precluding the use of animal models. While there have been a few studies of the URT microbiota associated with COVID, these have been largely exploratory studies that usually limited the description of taxa to the genus level, which is insufficient to detect IgA1 protease-producing species, and these were not specifically sought. However, some recent studies of the nasopharyngeal microbiota of patients with SARS-CoV-2 infection demonstrated statistically significant alterations in terms of richness (i.e., α- and β-diversity) and composition when compared with healthy subjects. These include increased proportions of bacteria that are known to produce IgA1 protease, that is, *S. pneumoniae*, certain other *Streptococcus* spp., *H. influenzae*, *Gemella*, and *Capnocytophaga*, (along with other species lacking this activity) (36, 37). Furthermore, two studies have reported an association of pneumococcal carriage detection and density with SARS-CoV-2, which suggest a synergistic relationship in the upper airway (36, 38). In a study of 76 children in rural Cote d’Ivoire, an association of URT infection including SARS-CoV-2 with colonization by *S. pneumoniae* or *H. influenzae* was noted (39). Thus, several studies suggest the possibility of an interaction between colonization with IgA1 protease-producing bacteria and URT viral infections, although they lack a focused mechanistic angle that might afford an explanation, and further studies in this direction are required. Our hypothesis offers one possible explanation that is amenable to experimental investigation.

## ADDITIONAL CONSIDERATIONS

The impact of IgA1 protease-producing bacteria might not be limited to SARS-CoV-2 infection, as it could possibly apply also to other infections such as influenza or respiratory syncytial virus, both of which are of current concern. Before influenza virus was discovered in 1933, influenza was often attributed to *H. influenzae*, which was isolated from many subjects infected with influenza in the 1918 epidemic, hence its species name. The question can therefore be asked whether *H. influenzae* might facilitate the acquisition and transmission of influenza virus by this mechanism, thereby giving rise to the erroneous idea that it causes influenza.

It might be argued that cleaving virus-neutralizing SIgA1 antibodies against viruses would confer no survival advantage to the IgA1 protease-producing bacteria, and would therefore not be functionally relevant to them. We think that is immaterial, as the facts that IgA1 protease activity has evolved independently in at least three or four lineages of bacteria, and that these enzymes are expressed constitutively, usually by all strains of each species, implies that they fulfil important roles in each organism’s lifestyle, regardless of their impact on other microbes (29, 30). Moreover, it is possible that by interfering with host defenses against other organisms, whether viral or bacterial, advantage might be obtained from disruption of the microbiota in the mucosal environment. Such advantages could include perturbation of host defenses as well as increasing the supply of nutrients resulting from mucosal damage.

## CONCLUDING REMARKS

Numerous diverse bacterial species produce IgA1 proteases that specifically cleave human IgA1 into Fabα and Fcα fragments, thereby compromising the biological functions of IgA (including SIgA) antibodies. These enzymes are generated constitutively by most strains of several distinct human mucosal pathogens and commensals, implying that they have significant impacts on human infections, especially of the mucosae where SIgA is the predominant form of antibody. However, relatively few examples of such impacts have been described (but see refs. 32, 33). The growing realization that SARS-CoV-2 is initially a mucosal infection of the URT, and that it induces mucosal IgA antibody responses that may be critical to the outcome of the initial infection, coupled with the likely presence of IgA1 protease-producing bacteria in the sites of initial infection, should stimulate investigation of the potential intersection of these two phenomena in the pathogenesis of COVID and its transmission within the community (1). Box 1 outlines some key studies to further evaluate and test the hypothesis.

BOX 1. **Key questions to evaluate and test the hypothesis**

- Determine the virus-neutralizing activity of Fabα fragments in comparison with intact mIgA and pIgA antibodies.
- Determine the presence of IgA1 protease-producing bacterial species in COVID-19 patients in comparison with control subjects.
- Determine the presence of characteristic Fabα fragments of IgA antibodies against the virus in saliva and nasal fluids of COVID-19 patients and control subjects, in relation to carriage of IgA1 protease-producing bacteria.

­
